# Single-Cell Sequencing of Glioblastoma Reveals Central Nervous System Susceptibility to SARS-CoV-2

**DOI:** 10.3389/fonc.2020.566599

**Published:** 2020-11-16

**Authors:** Bingshan Wu, Weihong Wang, Haopeng Wang, Quanli Zou, Benxia Hu, Lei Ye, Yangchun Hu, Yuhuan Xie, Nali Huang, Qing Lan, Hongwei Cheng, Jun Dong, Xingliang Dai

**Affiliations:** ^1^ Department of Neurosurgery, The First Affiliated Hospital of Anhui Medical University, Hefei, China; ^2^ Lab of Single Cell, Sinotech Genomics Co., Ltd., Shanghai, China; ^3^ UNC Neuroscience Center, University of North Carolina, Chapel Hill, NC, United States; ^4^ Department of Genetics, University of North Carolina, Chapel Hill, NC, United States; ^5^ Brain Tumor Lab, Department of Neurosurgery, The Second Afﬁliated Hospital of Soochow University, Suzhou, China

**Keywords:** severe acute respiratory syndrome coronavirus 2, central nervous system, single-cell transcriptome analysis, Angiotensin Converting Enzyme-2, glioblastoma multiform

## Abstract

**Background:**

Severe acute respiratory syndrome coronavirus 2 (SARS-CoV-2) caused the recent global COVID-19 outbreak, which led to a public health emergency. Entry of SARS-CoV-2 into human cells is dependent on the SARS-CoV receptor, angiotensin converting enzyme 2 (ACE2) receptor, and cathepsin. Cathepsin degrades the spike protein (S protein), which results in the entry of viral nucleic acid into the human host cell.

**Methods:**

We explored the susceptibility of the central nervous system (CNS) to SARS-CoV-2 infection using single-cell transcriptome analysis of glioblastoma.

**Results:**

The results showed that ACE2 expression is relatively high in endothelial cells (ECs), bone marrow mesenchymal stem cells (BMSCs), and neural precursor cells (NPCs). Cathepsin B (Cat B) and cathepsin (Cat L) were also strongly expressed in various cell clusters within the glioblastoma microenvironment. Immunofluorescence staining of glioma and normal brain tissue chips further confirmed that ACE2 expression co-localized with CD31, CD73, and nestin, which confirmed the susceptibility to SARS-CoV-2 of nervous system cells, including ECs, BMSCs, and NPCs, from clinical specimens.

**Conclusions:**

These findings reveal the mechanism of SARS-CoV-2 neural invasion and suggest that special attention should be paid to SARS-CoV-2–infected patients with neural symptoms, especially those who suffered a glioma.

## Introduction

New coronaviruses, such as severe acute respiratory syndrome coronavirus 2 (SARS-CoV-2), have previously caused pandemics worldwide because of their high pathogenicity, high infectivity, high lethality, and ability to cause multiple organ damage. SARS-CoV-2 was isolated from samples of human airway epithelial cells and verified through unbiased sequencing. SARS-CoV-2 is the seventh member of the coronavirus family that infects humans and it is the third coronavirus to emerge in the human population in the new century. SARS-CoV-2 differs from other beta-coronaviruses including SARS-CoV and MERS-CoV. People infected with SARS-CoV-2 experience SARS-CoV-like pneumonia, but with more severe symptoms. The most common symptoms are fever and cough, but the most characteristic symptom is respiratory distress. However, critically ill patients often also present with multiple organ dysfunction and disorders. The mortality rate is as high as 61.5% in critically ill patients (1). This infection has been named COVID-19 (2). More than 17 million people worldwide have been infected with SARS-CoV-2 to date, and there have been more than 680,000 deaths (https://covid19.who.int/, Aug 6, 2020).

In addition to upper respiratory tract infections, human coronaviruses like SARS-CoV have also invaded the central nervous system (CNS) (3). Given the high degree of genetic similarity between SARS-CoV and SARS-CoV-2, it is expected that SARS-CoV-2 may also cause neurological symptoms. In fact, many COVID-19-infected patients have presented with signs of neurologic symptoms like headache, nausea, and vomiting (4). These symptoms are strongly associated with poorer outcomes (5). Moreover, Moriguchi T. reported the first case of meningitis/encephalitis associated with SARS-CoV-2 infection in the cerebrospinal fluid (CSF) without a positive nasopharyngeal swab (6). Whether SARS-CoV-2 can invade the CSF or CNS of asymptomatic patients is unknown. Additionally, the susceptibility of human CNS cells to SARS-CoV-2 and its underlying pathogenic mechanisms are unclear.

Coronavirus entry into the host cell involves fusion of the virus to the cell membrane. This is mediated by the binding of viral spike (S) proteins to cellular receptors and the priming of relevant host cell proteases by S protein priming. SARS-CoV-2 recognizes the zinc peptidase angiotensin converting enzyme 2 (ACE2) receptor for cell entry (7). Meanwhile, it can use the endosomal cysteine proteases cathepsin B and L (CatB/L, gene CTSB/L) for S protein priming. The inhibition of both proteases is required for a robust blockade of viral entry (8). ACE2 is highly expressed in alveolar epithelial cells, heart, and kidney cells. However, ACE2 expression also occurs in the gastrointestinal tract, bone marrow, and brain (9). The distribution of ACE2 expression suggests that SARS-CoV-2 may also infect other tissues and organs by binding ACE2, which could lead to multiple organ damage.

It is important to outline the expression profile of ACE2 in the neural system to uncover the susceptibility of the neural system to SARS-CoV-2 infection. In this study, we characterized ACE2 expression and CatB/L localization in glioblastoma (GBM) tissues and revealed the possible susceptibility of the CNS to SARS-CoV-2 infection.

## Methods

### Single-Cell Sequencing for Whole Transcriptome Amplification

This study protocol was approved by the Ethical Committee on Clinical Study of our university, according to the Declaration of Helsinki. Four GBM tissue specimens and their adjacent brain tissues were collected from the First Affiliated Hospital of Anhui Medical University (Hefei, China). Written consent was obtained from all patients. The pathological diagnosis was confirmed by pathologists at our hospital. All tissue specimens were cryopreserved before testing.

To obtain the transcriptomic expression profile of single-cells from glioblastoma tissue and its adjacent normal tissue, a microwell-based BD Rhapsody system (BD, San Jose, CA) was used according to the manufacturer’s protocol. Briefly, glioblastoma tissue and adjacent tissue were enzymatically dissociated into a single cell solution. The samples were kept on ice until they were loaded into the BD Rhapsody system for single cell transcriptome isolation. The final library was generated using Microbead-captured single cell transcriptome as per the manufacturer’s protocol using the BD Rhapsody cDNA Kit (BD Biosciences, 633773) and the BD Rhapsody Targeted mRNA & AbSeq Amplification Kit (BD Biosciences, 633774). The library was sequenced in the X Ten instrument (Illumina, San Diego, CA) using the PE150 mode (Pair-End for 150-bp read).

### Single Cell RNAseq (scRNAseq) Analysis

Raw reads were processed to generate single-cell gene expression matrices through the BD Rhapsody Whole Transcriptome Assay Analysis Pipeline. Seurat (version 3.0.1) software was used for downstream clustering and visualization. In the filtering step, genes that were expressed in less than 0.1% of total cells were removed. Cells were also removed if there was more than 35% unique molecular identifiers (UMIs) that were derived from the mitochondrial genome. Canonical correlation analysis (CCA) integration was performed between two batches. In total, 12,118 cells collected from four patients were further analyzed.

The top 2000 highly variable features and the top 20 principal components were selected for further clustering analysis. Nineteen clusters were identified with the default resolution. To create a heatmap plot, the top 10 markers from each cluster were selected. A downstream pseudotime trajectory analysis was performed with Monocle2 software using default parameters. Gene Ontology (GO) and Kyoto Encyclopedia of Genes and Genomes (KEGG) functional enrichment analysis were performed using the clusterProfiler which was analyzed using the R package. GO and KEGG pathway enrichment analysis for differentially expressed genes (DEGs) was also performed using the clusterProfiler package. We also used clusterProfiler software to compare these gene clusters by their enriched biological processes, with a false discovery rate (FDR) <0.05.

### Immunohistochemistry (IHC) and Immunofluorescence Assay

Chips of human glioma tissue specimens that included 5 normal brain tissues, 13 low grade gliomas, and 19 high grade gliomas were analyzed to investigate the localization of target proteins using anti-human IgG antibody or control IgG. The primary antibodies used were rabbit anti-human ACE2 (Bioss, Beijing, China), mouse anti-human nestin antibody (Proteintech, Hubei, China), rabbit anti-cathepsin B antibody (Abcam, UK), rabbit anti-cathepsin L antibody (Abcam, UK), mouse anti-CD31 antibody (Abcam, UK), and mouse anti-CD73 monoclonal antibody (Santa Cruz, US). The IHC conditions for measuring ACE2 expression were pre-optimized on checkboards with multiple tissue samples. Briefly, paraffin-embedded tissue sections were blocked with 5% normal goat serum for 30 min. After deparaffinization, samples were rehydrated and endogenous peroxidase was blocked. The slides were then incubated with the primary antibody at a dilution of 1:50 for 20 min at room temperature (or at 4°C overnight in a humid chamber). Subsequently, the sections were incubated for 30 min at room temperature with CY3-conjugated goat anti-rabbit IgG secondary antibody (Abcam, UK), FITC-conjugated goat anti-mouse IgG (Abcam, UK), or goat anti-rabbit IgG (Alexa Fluor 488, Abcam, UK). The cell nuclei were stained using 4′, 6-diamidino-2-phenylindole (DAPI; Beyotime, China). The negative control was a stain of cells that were not incubated with the primary antibodies. Cells were washed with PBS three times prior to mounting and were visualized with a fluorescence microscope (Motic, Xiamen, China).

### Cell Culture

The human glia cell line HA1880 was purchased from BeNa Culture Collection (BNCC, Beijing). U87 and U251 were obtained from the Chinese Academy of Sciences, ShangHai Cellbank (Shanghai, China). Glioma cell line SHG44 and glioma stem-like cell line SU3 were isolated by our lab (10, 11). U251 stem-like cells (U251s) were enriched and isolated from U251 cell line *via* cultivating with stem cell culture medium. The cells (HA1800, U87, U251 and SHG44) were maintained in Dulbecco’s modified Eagle’s medium (Invitrogen, Valencia, CA) supplemented with 10% fetal bovine serum (Invitrogen) in a humidified incubator with 5% CO2 at 37°C, and were passaged twice weekly. Glioma stem-like cells U251s and SU3 were cultured in Dulbecco’s modified Eagle’s medium DMEM/F12 medium (Gibco, USA) containing 20 ng/ml basic fibroblast growth factor (Gibco), 20 ng/ml epidermal growth factor (Gibco), B27 supplement (50×), 2 mM l-glutamine, MEM vitamin solution and 100 mM sodium pyruvate (100×) (Gibco).

### Western Blotting

Whole-cell extracts were prepared using ProteoJET Mammalian Cell Lysis Reagent (Fermentas, Burlington, Canada) supplemented with protease and phosphatase inhibitors (Fermentas) according to the manufacturer’s instructions. Protein (20–40 mg) was separated by sodium dodecyl sulfate–polyacrylamide gel electrophoresis in 8%–10% gels and transferred to polyvinylidene difluoride membranes (Millipore, Billerica, MA). The blots were blocked for 1 h at room temperature with 5% bovine serum albumin (Sigma) in Tris-buffered saline/0.1% Tween-20. Next, the blots were probed with anti-ACE2 (Bioss, Beijing, China) or anti-GAPDH (Proteintech, Wuhan, China) antibodies overnight at 4°C. The blots were then incubated with corresponding horseradish peroxidase- conjugated GAPDH (Zsbio, Beijing) for 1 h at room temperature. After additional washes, signals were detected using SuperSignal ECL (Pierce, Rockford, IL).

## Results

### scRNA-Seq Profiling Identifies Cell Types and Sub-Cell Clusters in GBM and Brain Cells

**Figure 1:**
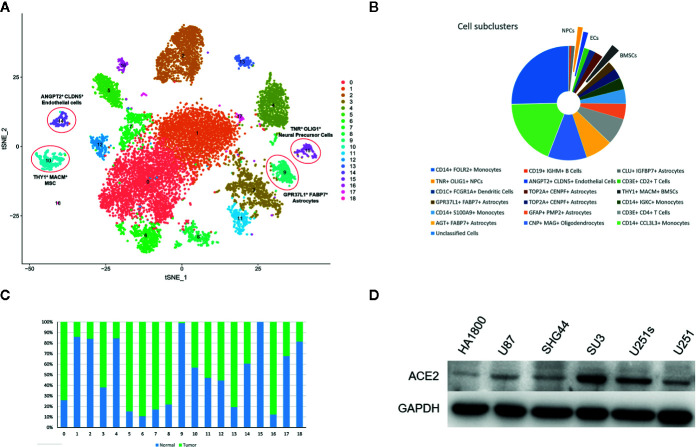
Single cell atlas of tested cells. **(A)**. tSNE plots showing 12,118 normal and cancer cells. Cells were classified into 19 cell clusters and labeled with different colors. **(B)** Relative proportions of the 19 cell clusters. **(C)** Relative percentages of normal and cancer cells in each cell cluster. **(D)** ACE2 expression level in glial, glioma and glioma stem-like cell lines.

We profiled four GBM samples and four normal tissues that were collected from an area adjacent to the tumor using the BD Rhapsody system. After dissociation, dead cells and enucleated cellular debris were discarded. Live intact cells were isolated using fluorescence-activated cell sorting (FACS), computational cell selection, and filtering. After scRNA-seq analysis, a total of 12,118 cells were isolated and about 500 genes and UMIs were detected per cell. After removing the batch effect, cells were sorted into 19 clusters (**Figure 1A**). A heatmap of the main cell gene markers across the different cell types was created (**Figure S1**). Ten subtypes of cells were discovered from the 19 cell clusters: monocytes (51.5%), oligodendrocytes (11.0%), astrocytes (19.7%), T-cells (9.1%), bone marrow stromal cells (2.4%), dendritic cells (1.8%), endothelial cells (1.4%), neural precursor cells (1.3%), B lymphocytes (0.6%), and unclassified cells (1.0%) (**Figure 1B**). Fractional differences between normal and cancer cells in each cell cluster were evaluated (**Figure 1C**). ACE2 expression at protein level in glial/glioma cell lines was detected by Western Blot analyses, which demonstrate that ACE2 expressed at a higher level in glioma stem-like cell lines than in glioma cell lines. However, ACE2 expressed relatively low in glia cell line HA1880 (**Figure 1D**).

### ACE2 Is Expressed at a High Level in Endothelial Cells, Bone Marrow Mesenchymal Stem Cells (BMSCs), and Neural Precursor Cells (NPCs)

**Figure 2:**
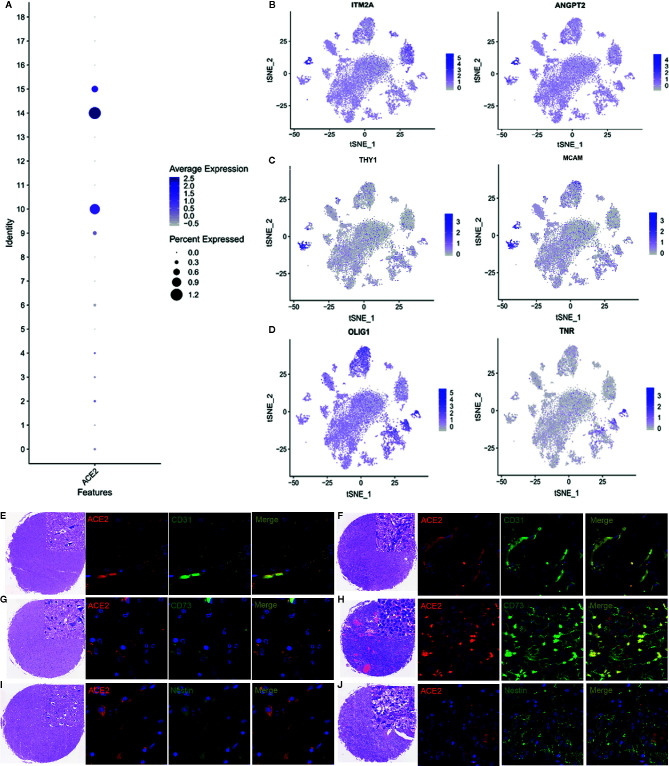
ACE2 expression in endothelial cells, BMSCs, and NPCs. **(A)** Relative expression of ACE2 in clusters 10 (BMSCs), 14 (endothelial cells), and 15 (NPCs). **(B)** Cell marker expression of BMSCs. **(C)** Cell marker expression of endothelial cells. **(D)** Cell marker expression of NPCs. (E–J) Immunofluorescence analysis of ACE2 expression in endothelial cells, BMSCs, and neural progenitors. **(E, G, I)** Expression of ACE2 in normal tissue. **(F, H, J)** Expression of ACE2 in tumor samples. CD31, CD73, and nestin served as marker genes for all cells. BMSC: bone marrow stem cells, NPC: neural precursor cells, ACE2: angiotensin converting enzyme 2 receptor.

To investigate whether SARS-CoV-2 could infect the identified cell subtypes, we examined ACE2 expression in each cell type. Although ACE2 may be expressed in all cell types (**Figure S2**), ACE2 expression was higher in endothelial cells, BMSCs, and NPCs (**Figure 2A**). There was high expression of marker genes in the corresponding cell-type on the basis of t-distributed stochastic neighbor embedding (t-SNE) analysis (**Figures 2B–D**). CD31, CD73, and nestin were used to identify endothelial cells, BMSCs, and neural precursor cells, respectively (**Figures 2E–J**). Colocalization analysis confirmed that ACE2 was highly expressed in those cell types, indicating that SARS-CoV-2 can infect endothelial cells, BMSCs, and neural progenitors (**Figures 2E–J**). Compared to normal tissue (**Figures 2E, G, I**), ACE2 expression was higher in tumor samples (**Figures 2F, H, J**) .

### CTSB and CTSL Expression in Cell Clusters

**Figure 3:**
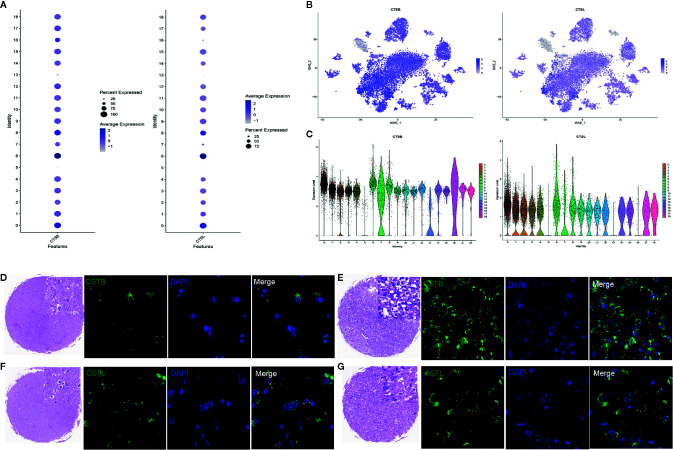
CTSB and CTSL expression in cell clusters. **(A)** Relative expression of CSTB and CSTL in all cell clusters. **(B)** CSTB and CSTL. **(C)** CSTB and CSTL expression. **(D, F)** CSTB and CSTL expression in normal tissue. **(E, G)** CSTB and CSTL expression in tumor samples. Blue: represents nucleus stained with DAPI, Green: represents cells stained with CSTB and CSTL.

CTSB and CTSL play an important role during coronavirus cell invasion. Interestingly, CTSB and CTSL were highly expressed in endothelial cells, BMSCs, and NPCs (**Figures 3A–C**). Consistent with scRNA-seq, immunofluorescence staining further demonstrated that CTSB/L were upregulated in tumors compared to normal tissues (**Figures 3D–G**).

### A Potential Mechanism for Viral Neural Invasion

**Figure 4:**
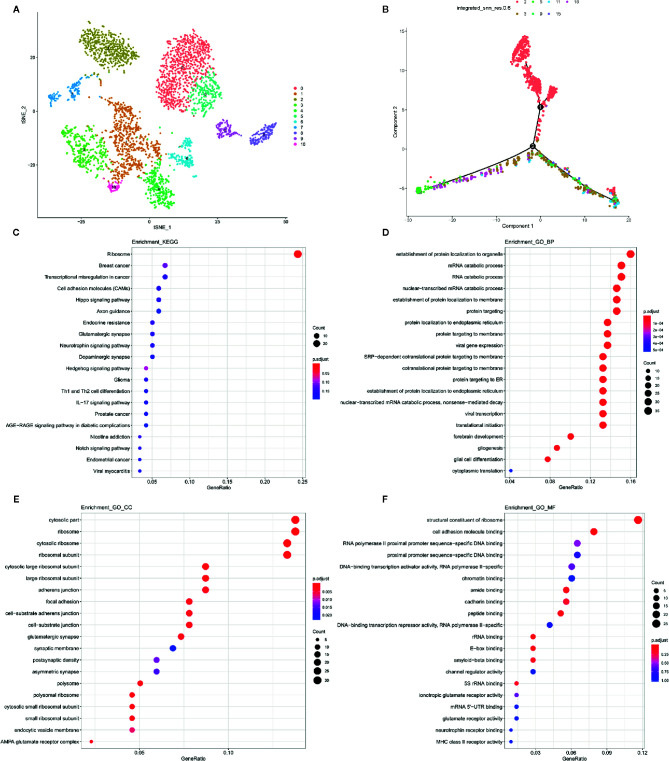
Potential mechanism of viral invasion of the neural system. **(A)** UMAP of non-immune cell sub-clusters. **(B)** Pseudotime trajectory analysis of non-immune cells. **(C)** KEGG enrichment analyses of NPC cells. **(D)** Related biological processes of GO analysis of NPC cells. **(E)** Cellular components of GO analysis of NPC cells. **(F)** Molecular Function of GO analysis of NPC cells. Dots represent term enrichment with color coding: red indicates high enrichment, blue indicates low enrichment. The sizes of the dots represent the percentage of each row. NPC, neural precursor cells; GO, gene ontology; KEGG, Kyoto Encyclopedia of Genes and Genomes.

After exclusion of immune cell clusters, cells were subdivided into 10 clusters (**Figure 4A**). A heatmap of the cellular markers is shown in **Figure S3**. Pseudotime trajectories were applied to the 10 clusters to investigate cellular dynamic processes. Human neural precursor cells (hNPCs) constituted cluster 6 (**Figure 4B**). Based on KEGG enrichment analysis, differentially expressed genes in the blue module were enriched in the ribosome (**Figure 4C**). As expected, GO analysis found that some genes may be involved in viral gene expression and transcription (**Figures 4D–F**). It is suggested that SARS-CoV-2 may cause pathogenic outcomes in the nervous system *via* primitive infection of NPC cells.

## Discussion

Entry of coronavirus into the host cell is a multi-step process mediated by its S protein. The S1 subunit of the S protein binds a receptor on the host cell surface, while subunit S2 fuses the host and viral membranes to allow the viral genome to enter host cells (12). Host cell entry of SARS-CoV-2 depends on the SARS-CoV receptor ACE2, suggesting that the virus might target a similar spectrum of cells as SARS-CoV.

SARS-CoV genome sequences were detected in the brain of SARS victims. Pathologic changes such as edema and scattered red degeneration of the neurons were also present in the brains of these confirmed SARS cases (13). The functional receptor ACE2 is generally expressed in various organs including the brain (9), which suggests that SARS-CoV-2 may also invade the CNS through this possible route.

We investigated the expression of ACE2 in the brain using the scRNA transcriptome method. The optimal function of the CNS depends on the maintenance of its milieu. The blood-brain barrier (BBB) serves as the first line of defense that prevents pathogens from entering the brain. It is highly complex and composed of cerebral microvascular endothelium, astrocytes, pericytes, and a lamina basalis. Viral pathogens can breach protective barriers in a number of ways. One means for viral entry into the CNS is through the CNS endothelium.

In many cases, infection promotes chemokine secretion by endothelial cells, which increases vascular permeability and permits viruses to bypass the first layer of the BBB. Moreover, viruses also use proteins expressed by endothelial cells to bind and enter these cells (14). Although coronavirus infections are restricted to the airways, in rare conditions they may enter through the epithelial barrier and reach the CNS. This was also suggested for other respiratory viruses including respiratory syncytial virus (RSV), Nipah virus, and influenza virus (3). We found that ACE2 was highly expressed in brain endothelial cells. SARS-CoV-2 uses the SARS-CoV receptor ACE2 for host cell entry (8). Therefore, it is reasonable that SARS-CoV-2 could use the ACE2 receptor for cellular entry into the CNS by infecting the endothelium.

Another critical route of viral spread to the CNS is through the peripheral nerves or olfactory sensory neurons. A virus can infect neurons in the periphery and then use active transport within those cells to gain CNS access. The differential expression of viral receptors on either sensory or motor neurons can dictate the type of peripheral nerve ending that a particular neurotropic virus will target. Although the olfactory bulb is highly efficient at controlling neuroinvasion, several viruses can enter the CNS through the olfactory route (3). SARS-CoV infects the respiratory tract after intranasal infection in mice and the extrapulmonary virus can then spread to the brain (15). An experiment investigating ACE2 transgenic mice showed that SARS-CoV enters the brain primarily *via* the olfactory bulb, and infection results in trans-neuronal spread to other connected areas of the brain, especially the medulla. The medulla is where the cardiorespiratory centers are located, and medulla infection with SARS-CoV was the major cause of death in animals (16).

**Figure 5:**
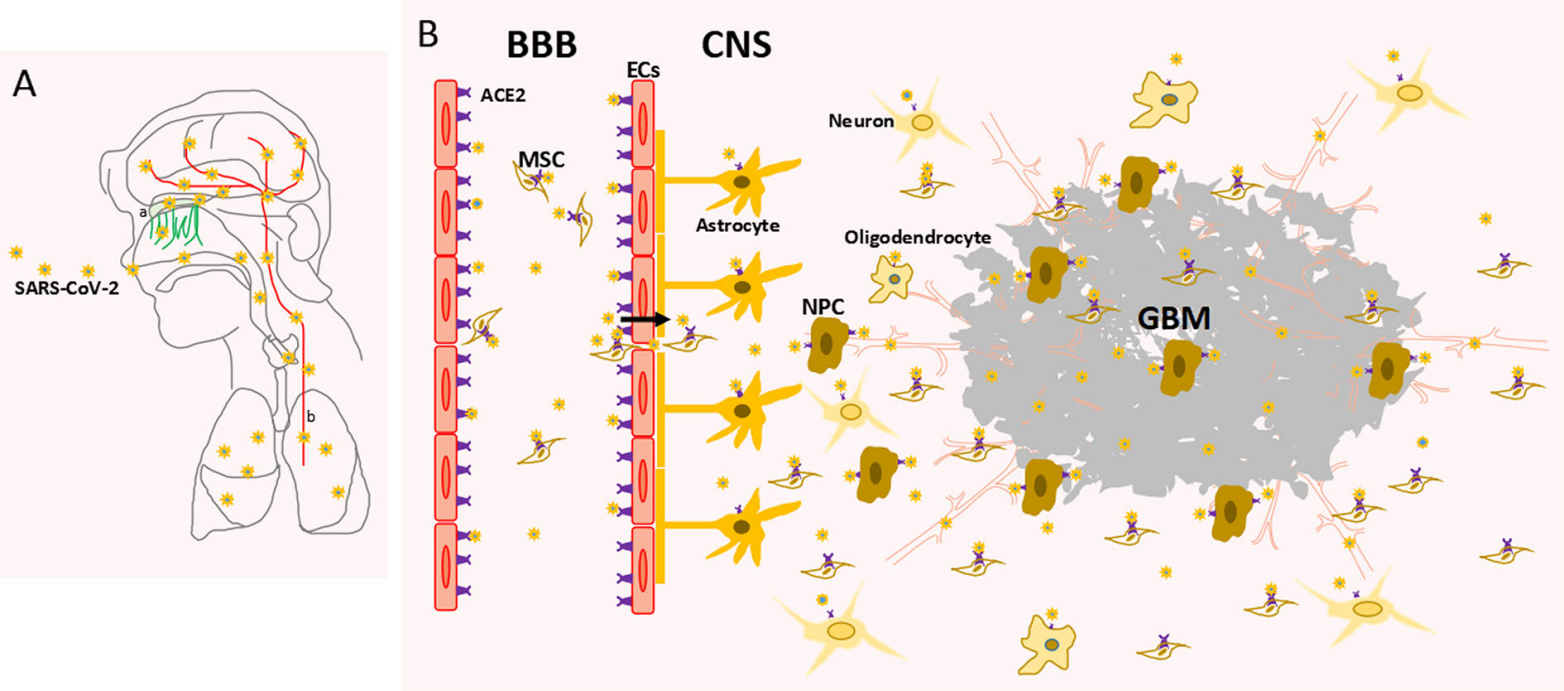
Schematic diagram of CNS invasion by SARS-CoV-2 **(A)** SARS-CoV-2 invades the CNS through the olfactory nerve (a) and blood route (b). **(B)** SARS-CoV-2 invades the CNS through blood-brain barrier (BBB). ACE2, angiotensin converting enzyme 2 receptor; NPC, neural precursor cell; EC, endothelial cell; MSC, mesenchymal stem cell; GBM, glioblastoma.

The olfactory bulb and cell migration from the subventricular zone (SVZ) along the rostral migratory stream to the olfactory bulb are a source of NPCs in mammalian animals (17). NPCs continually self-renew and differentiate into terminal glial and neural cells, which implies that infected NPCs could be a probable source of CNS infection. For glioma patients, glioma stem-like cells are also a potential cell subset that is more susceptible to the SARS-CoV-2 than glioma cells since glioma stem-like cells possess a higher ACE2 expression level. Additionally, GO and KEGG enrichment analysis revealed that NPCs are vulnerable to virus infection. Although NPCs have not been reported as being directly involved in SARS-CoV-2 infection, a recent study has shown that loss of sense of smell was extensively associated with a COVID-19–positive infection (18). This provides evidence that a loss of olfactory sensation mediated by viral infection indicates a neurotrophic property of SARS-CoV2. SARS-CoV-2 may invade the CNS through ACE2 binding in the olfactory bulb and could infect the brain *via* the binding to ACE2 on NPCs (**Figure 5A**).

The pathological features of COVID-19 are similar to those of SARS and MERS coronavirus infection. Exaggerated cytokine and chemokine responses play an important role in the immune response during SARS-CoV infections. While SARS-CoV primarily infects airway and alveolar epithelial cells, infection of hematopoietic cells such as dendritic cells, monocyte-macrophages, and other peripheral blood mononuclear cell (PBMC)-derived cells is futile. SARS-CoV infection of these cells upregulates the expression of various cytokines and chemokines, which results in a dysregulated innate immune response called a cytokine storm (19). This cytokine storm depends on the rapid expansion of hematopoietic stem cells and their differentiation into all mature blood cell subtypes in response to stress stimuli. It has been found that GSC niches are being formed in glioblastoma as a copy of HSC niches in bone marrow (20). The bone marrow microenvironment, and particularly BMSCs, provide a special niche and secrete soluble factors that support the survival, differentiation, and proliferation of hematopoietic cells.

Mitochondrial transfer from stromal cells into blood stem cells is required for the rapid generation of leukocytes in response to pathogen infection (21). A previous study showed that bone marrow stromal cells are a novel target for RSV infection. RSV infection causes important structural and functional changes in a cell population that affects hematopoietic and immune functions (8). Our study showed that BMSCs express ACE2, which indicates that SARS-CoV-2 might infect BMSCs, which affects the modulation of the immune response by hematopoietic cells. Future prophylactic and management strategies for COVID-19 should also address the treatment of possible extrapulmonary sites of viral replication. Furthermore, in this pathological situation, BMSCs were recruited across the BBB and suggest that the virus may enter the CNS *via* a “Trojan horse” mechanism. In this case, infected leukocytes could carry the pathogen from the blood across the BBB (**Figure 5B**).

Cathepsins are a diverse group of acid-activated cysteine proteases located within endosomes and lysosomes. CTSB protein expression was found to be associated with GBM cells, macrophages/microglia cells and endothelial cells in glioma tissue sections (22). CTSL activity is required for infection by several viruses like SARS and MERS that enter cells *via* the endosomal route (23, 24). CTSL proteolysis induced conformational changes in S glycoprotein and activated membrane fusion within endosomes, which directly modulated the fusion activity of SARS-CoV S protein. A specific and irreversible inhibitor of CTSL significantly inhibited SARS S protein-mediated cell entry, suggesting that CTSL is a therapeutic target for antiviral intervention. We found that CTSB and CTSL were co-expressed with ACE2 in several brain cell types. CTSL may activate the membrane fusion of SARS-CoV-2 S and facilitate viral binding to ACE2. In other words, CTSL utilizes the enzymatic activity of the cysteine protease cathepsin L to infect ACE2-expressing cells (25).

Since the samples in this study could only be harvested from glioma patients but not healthy candidates, the results of this study are limited. However, we still provided evidence that SARS-CoV-2 may invade the CNS *via* many susceptible cells. Furthermore, a recent study showed that patients with cancer located in the epicenter of a viral epidemic had a higher risk of SARS-CoV-2 infection compared to the community (26). According to the data, aggressive steps should be taken to protect patients with glioma during the COVID-19 pandemic to mitigate the neurological risks of SARS-CoV-2 infection.

## Conclusion

Our study verified that ACE receptors were expressed in CNS cells. This indicated that the CNS has an intrinsic susceptibility to SARS-CoV-2 infection. Patients with glioblastoma exhibited relatively high ACE2 expression and may be more susceptible to progression to a more severe infection. Our study further clarified that the mechanism of SARS-Cov-2 CNS invasion might be through the binding of the ACE2 receptor and that CTSB/L may facilitate this process. The findings of this study show the neurotropic potential of the SARS-CoV-2 virus and may provide guidance on how to clinically treat neural symptoms in patients with COVID-19.

## Data Availability Statement

The original contributions presented in the study are publicly available. This data can be found here: https://www.ncbi.nlm.nih.gov/geo/query/acc.cgi?acc=GSE157424.

## Ethics Statement

The studies involving human participants were reviewed and approved by Ethical Committee on Clinical Study of the First Affiliated Hospital of Anhui Medical University. The patients/participants provided their written informed consent to participate in this study.

## Author Contributions

HC, JD, and XD conceived and planned the study. BW, WW, and HW performed the experiments. LY and YH contributed to sample preparation. QZ, BH, and NH analyzed the data. YX and QL contributed to the interpretation of the results. All authors contributed to the article and approved the submitted version. BW and XD wrote the paper with input from all authors.

## Funding

This study was supported by the National Natural Scientific Foundation of China (No. 81702457), China National Nuclear Corporation Youth Innovation Team Project (No. 14070) and Suzhou Science and Technology Project (No. SYS201723).

## Conflict of Interest

Authors QZ and NH were employed by the company Sinotech Genomics Co. Ltd.

The remaining authors declare that the research was conducted in the absence of any commercial or financial relationships that could be construed as a potential conflict of interest.

## Supplementary Material

Supplementary Figure S1 Gene marker heatmap. Heatmap showing the marker genes of 19 cell clusters (highlighted).

Supplementary Figure S2 Expression of ACE2 in 19 cell clusters **(A)** tSNE; **(B)** UMAP.

Supplementary Figure S3 Marker genes heatmap of non-immune cells Heatmap showing the marker genes of 19 non-immune cell clusters which are highlighted.

## Abbreviations

SARS-CoV-2, severe acute respiratory syndrome coronavirus 2; ACE2, angiotensin converting enzyme 2; CNS, central nervous system; ECs, endothelial cells; BMSCs, bone marrow mesenchymal stem cells; NPCs, neural precursor cells; CSF, cerebrospinal fluid; GBM, glioblastoma; BBB, blood-brain barrier; CatB/L, cathepsin B and L.

## References

[1] Yang X Yu Y Xu J Shu H Xia J Liu H Clinical course and outcomes of critically ill patients with SARS-CoV-2 pneumonia in Wuhan, China: a single-centered, retrospective, observational study. Lancet Respir Med (2020) 8(5):475–81. 10.1016/S2213-2600(20)30079-5 PMC7102538 32105632

[2] Zhu N Zhang D Wang W Li X Yang B Song J A Novel Coronavirus from Patients with Pneumonia in China, 2019. N Engl J Med (2020) 382(8):727–33. 10.1056/NEJMoa2001017 PMC7092803 31978945

[3] Desforges M Le Coupanec A Dubeau P Bourgouin A Lajoie L Dube M Human Coronaviruses and Other Respiratory Viruses: Underestimated Opportunistic Pathogens of the Central Nervous System? Viruses (2019) 12(1):14. 10.3390/v12010014 PMC7020001 31861926

[4] Xu XW Wu XX Jiang XG Xu KJ Ying LJ Ma CL Clinical findings in a group of patients infected with the 2019 novel coronavirus (SARS-Cov-2) outside of Wuhan, China: retrospective case series. BMJ (2020) 368:m606. 10.1136/bmj.m606 32075786 PMC7224340

[5] Chen T Wu D Chen H Yan W Yang D Chen G Clinical characteristics of 113 deceased patients with coronavirus disease 2019: retrospective study. BMJ (2020) 368:m1091. 10.1136/bmj.m1091 32217556 PMC7190011

[6] Moriguchi T Harii N Goto J Harada D Sugawara H Takamino J A first case of meningitis/encephalitis associated with SARS-Coronavirus-2. Int J Infect Dis (2020) 94:55–8. 10.1016/j.ijid.2020.03.062 PMC7195378 32251791

[7] Letko M Marzi A Munster V Functional assessment of cell entry and receptor usage for SARS-CoV-2 and other lineage B betacoronaviruses. Nat Microbiol (2020) 5(4):562–9. 10.1038/s41564-020-0688-y PMC7095430 32094589

[8] Hoffmann M Kleine-Weber H Schroeder S Kruger N Herrler T Erichsen S SARS-CoV-2 Cell Entry Depends on ACE2 and TMPRSS2 and Is Blocked by a Clinically Proven Protease Inhibitor. Cell (2020) 181(2):271–80.e8. 10.1016/j.cell.2020.02.052 PMC7102627 32142651

[9] Hamming I Timens W Bulthuis ML Lely AT Navis G van Goor H Tissue distribution of ACE2 protein, the functional receptor for SARS coronavirus. A first step in understanding SARS pathogenesis. J Pathol (2004) 203(2):631–7. 10.1002/path.1570 PMC7167720 15141377

[10] Dai X Wang Y Dong X Sheng M Wang H Shi J Downregulation of miRNA-146a-5p promotes malignant transformation of mesenchymal stromal/stem cells by glioma stem-like cells. Aging (2020) 12(10):9151–72. 10.18632/aging.103185 PMC7288935 32452829

[11] Li R Tang D Zhang J Wu J Wang L Dong J The temozolomide derivative 2T-P400 inhibits glioma growth via administration route of intravenous injection. J Neuro-oncol (2014) 116(1):25–30. 10.1007/s11060-013-1255-7 24065569

[12] Li F Structure, Function, and Evolution of Coronavirus Spike Proteins. Annu Rev Virol (2016) 3(1):237–61. 10.1146/annurev-virology-110615-042301 PMC5457962 27578435

[13] Gu J Gong E Zhang B Zheng J Gao Z Zhong Y Multiple organ infection and the pathogenesis of SARS. J Exp Med (2005) 202(3):415–24. 10.1084/jem.20050828 PMC2213088 16043521

[14] McGavern DB Kang SS Illuminating viral infections in the nervous system. Nat Rev Immunol (2011) 11(5):318–29. 10.1038/nri2971 PMC5001841 21508982

[15] McCray PB Jr. Pewe L Wohlford-Lenane C Hickey M Manzel L Shi L Lethal infection of K18-hACE2 mice infected with severe acute respiratory syndrome coronavirus. J Virol (2007) 81(2):813–21. 10.1128/JVI.02012-06 PMC1797474 17079315

[16] Netland J Meyerholz DK Moore S Cassell M Perlman S Severe acute respiratory syndrome coronavirus infection causes neuronal death in the absence of encephalitis in mice transgenic for human ACE2. J Virol (2008) 82(15):7264–75. 10.1128/JVI.00737-08 PMC2493326 18495771

[17] Martin LJ Katzenelson A Koehler RC Chang Q The olfactory bulb in newborn piglet is a reservoir of neural stem and progenitor cells. PLoS One (2013) 8(11):e81105. 10.1371/journal.pone.0081105 24278384 PMC3836747

[18] Yan CH Faraji F Prajapati DP Boone CE DeConde AS Association of chemosensory dysfunction and Covid-19 in patients presenting with influenza-like symptoms. Int Forum Allergy Rhinol (2020) 10(7):806–13. 10.1002/alr.22579 PMC7262089 32279441

[19] Channappanavar R Perlman S Pathogenic human coronavirus infections: causes and consequences of cytokine storm and immunopathology. Semin Immunopathol (2017) 39(5):529–39. 10.1007/s00281-017-0629-x PMC7079893 28466096

[20] Hira VVV Breznik B Vittori M Loncq de Jong A Mlakar J Oostra RJ Similarities Between Stem Cell Niches in Glioblastoma and Bone Marrow: Rays of Hope for Novel Treatment Strategies. J Histochem Cytochem (2020) 68(1):33–57. 10.1369/0022155419878416 31566074 PMC6931169

[21] Mistry JJ Marlein CR Moore JA Hellmich C Wojtowicz EE Smith JGW ROS-mediated PI3K activation drives mitochondrial transfer from stromal cells to hematopoietic stem cells in response to infection. Proc Natl Acad Sci U S A (2019) 116(49):24610–9. 10.1073/pnas.1913278116 PMC6900710 31727843

[22] Breznik B Limbaeck Stokin C Kos J Khurshed M Hira VVV Bosnjak R X and K expression in peri-arteriolar glioblastoma stem cell niches. J Mol Histol (2018) 49(5):481–97. 10.1007/s10735-018-9787-y PMC6182580 30046941

[23] Qian Z Dominguez SR Holmes KV Role of the spike glycoprotein of human Middle East respiratory syndrome coronavirus (MERS-CoV) in virus entry and syncytia formation. PLoS One (2013) 8(10):e76469. 10.1371/journal.pone.0076469 24098509 PMC3789674

[24] Simmons G Gosalia DN Rennekamp AJ Reeves JD Diamond SL Bates P Inhibitors of cathepsin L prevent severe acute respiratory syndrome coronavirus entry. Proc Natl Acad Sci U S A (2005) 102(33):11876–81. 10.1073/pnas.0505577102 PMC1188015 16081529

[25] Huang IC Bosch BJ Li F Li W Lee KH Ghiran S SARS coronavirus, but not human coronavirus NL63, utilizes cathepsin L to infect ACE2-expressing cells. J Biol Chem (2006) 281(6):3198–203. 10.1074/jbc.M508381200 PMC8010168 16339146

[26] Yu J Ouyang W Chua MLK Xie C SARS-CoV-2 Transmission in Patients With Cancer at a Tertiary Care Hospital in Wuhan, China. JAMA Oncol (2020) 6(7):1108–10. 10.1101/2020.02.22.20025320 PMC7097836 32211820

