# Supplementary figures and images for: Single-Cell Sequencing of Glioblastoma Reveals Central Nervous System Susceptibility to SARS-CoV-2
Source: Front Oncol. 2020 Nov 16;10:566599. doi: 10.3389/fonc.2020.566599 (PMC7703438; doi:10.3389/fonc.2020.566599)

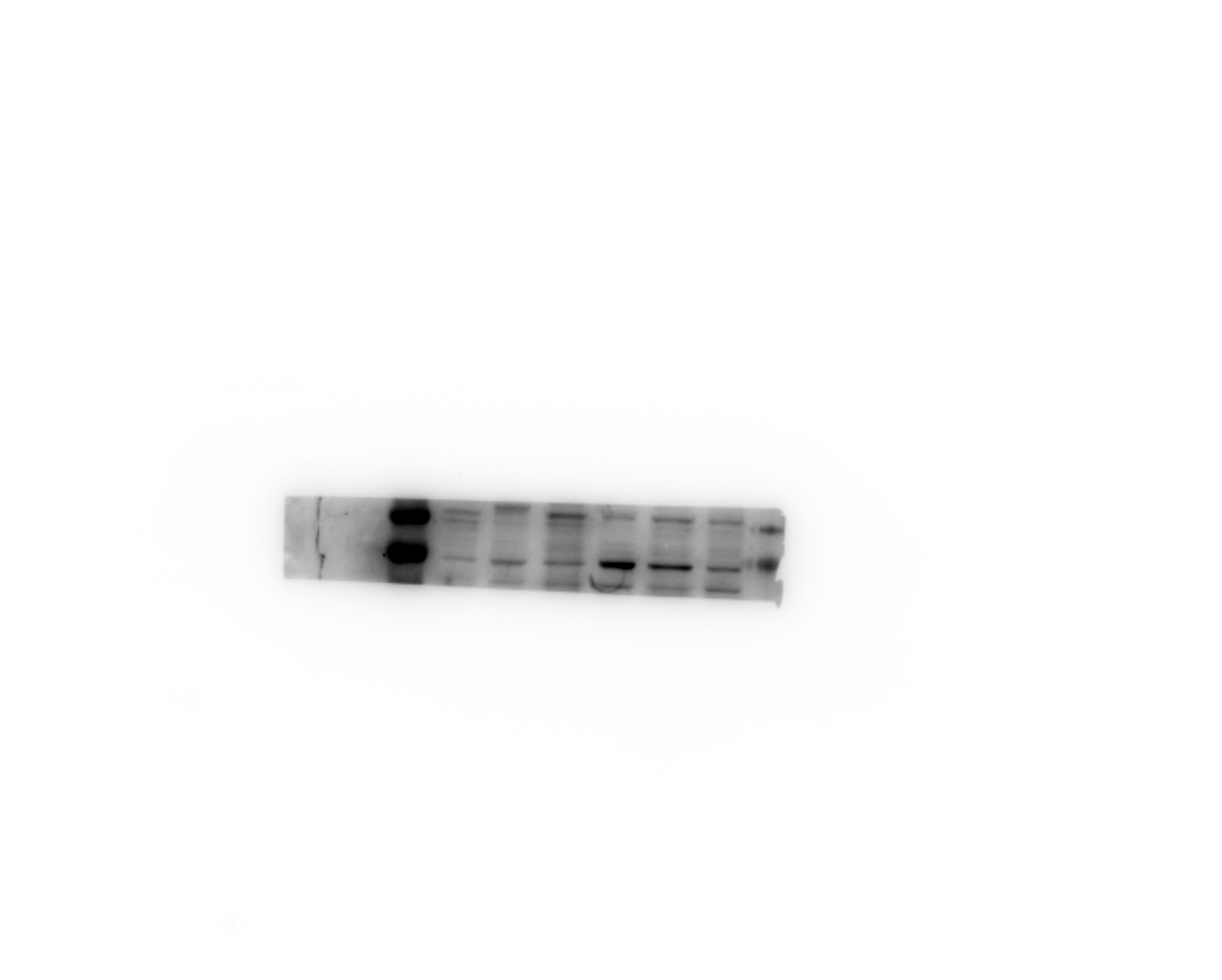

Supplement: Supplementary file 1 [file DataSheet_1.zip › ACE-2 (2).tif]

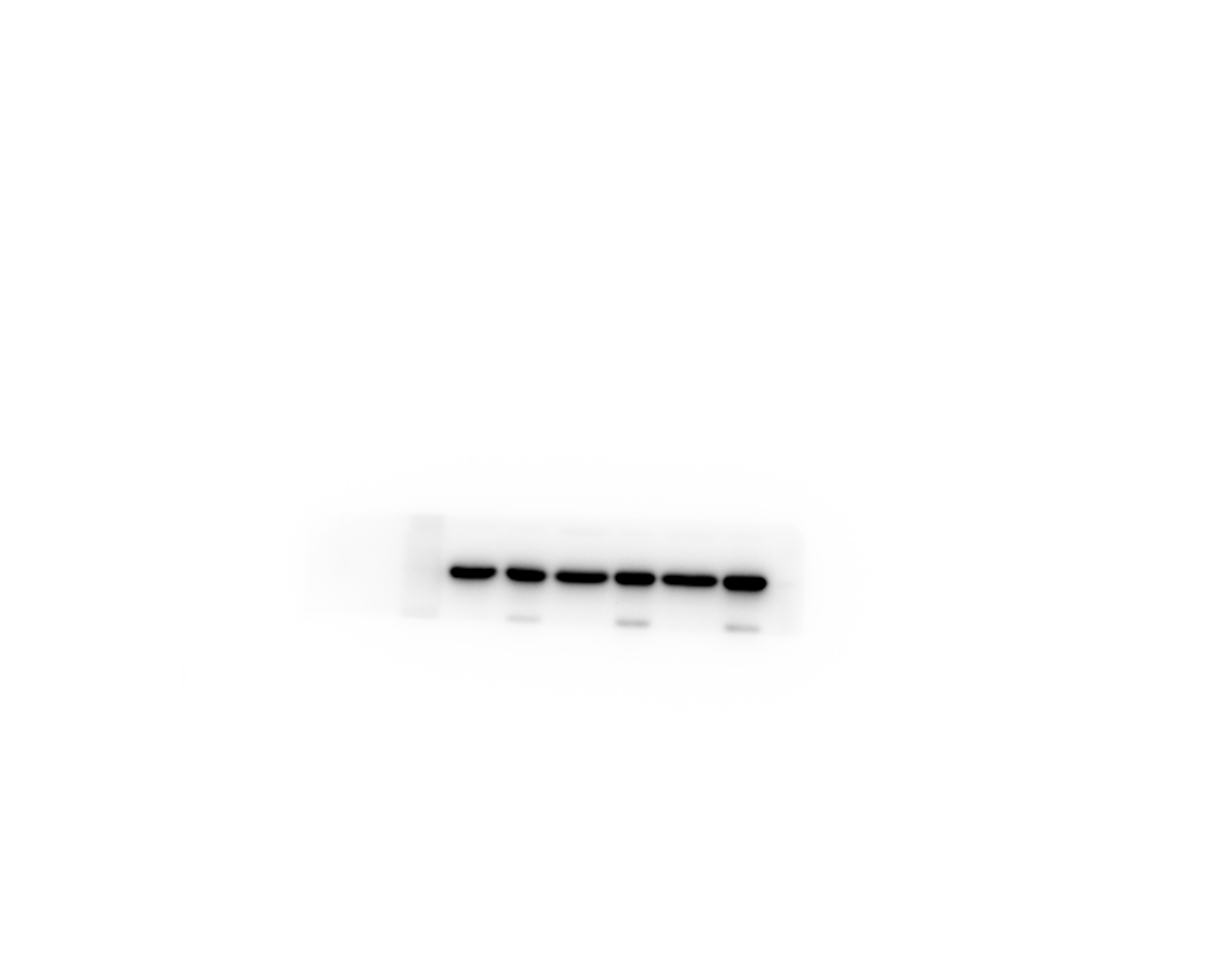

Supplement: Supplementary file 1 [file DataSheet_1.zip › GAPDH (1).tif]

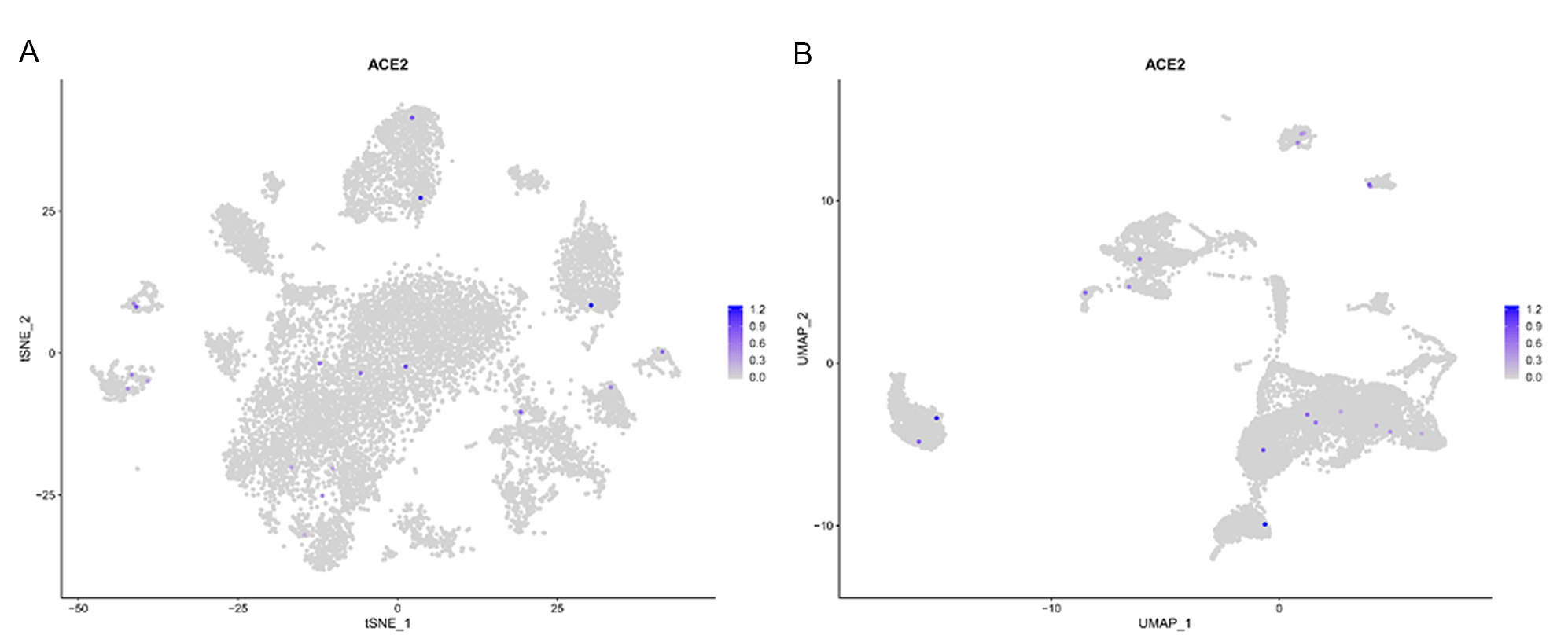

Supplement: Supplementary Figure S2 — Expression of ACE2 in 19 cell clusters (A) tSNE; (B) UMAP. [file Image_2.tif]
